# Personalized Diagnosis and Functional Impact of Vestibular Migraine in Women Aged 20–50: Cross-Sectional Analysis from Neurotology Clinic

**DOI:** 10.3390/jpm15080378

**Published:** 2025-08-14

**Authors:** Khalid A. Alahmari, Sarah Alshehri

**Affiliations:** 1 A Physical Therapy Program, Department of Medical Rehabilitation Sciences, College of Medical Applied Sciences, King Khalid University, Abha 61421, Saudi Arabia; kahmarie@kku.edu.sa; 2 Otolaryngology, Head and Neck Surgery, Department of Surgery, College of Medicine, King Khalid University, Abha 61421, Saudi Arabia

**Keywords:** vestibular migraine, dizziness, functional impairment, DHI, WPAI, quality of life

## Abstract

**Background/Objectives**: Vestibular migraine is a frequently underdiagnosed cause of dizziness in adult females, often overlapping clinically with other vestibular and neurological conditions. Despite its recognition in diagnostic criteria, limited data exist on its prevalence and functional impact in women presenting with dizziness in clinical settings. This study assesses the frequency and diagnostic rate of vestibular migraine among females aged 20–50 years presenting with dizziness and evaluates its impact on quality of life and work productivity. **Methods**: A cross-sectional study was conducted, which included 196 female patients reporting dizziness who were evaluated. Vestibular migraine was diagnosed using ICHD-3 criteria. Functional impact was assessed using the Dizziness Handicap Inventory (DHI) and Work Productivity and Activity Impairment (WPAI) questionnaire. Group comparisons and regression analyses were conducted using SPSS version 24. **Results**: Vestibular migraine was diagnosed in 84 participants, yielding a prevalence rate of 42.86% (95% CI: 36.13–49.86%). Compared to non-migraine participants, those with vestibular migraine had longer dizziness duration (37.62 ± 11.34 vs. 24.58 ± 10.49 min, *p* = 0.032), higher DHI (58.34 ± 15.62 vs. 32.76 ± 14.83, *p* < 0.001) and WPAI scores (42.19 ± 13.45 vs. 23.47 ± 12.90, *p* < 0.001), and more missed workdays. Regression analysis identified vestibular migraine, poor sleep, anxiety/depression, and BMI as significant predictors of work impairment. **Conclusions**: Vestibular migraine is a prevalent and functionally disabling condition among women with dizziness, underscoring the importance of systematic diagnosis and multidisciplinary management.

## 1. Introduction

Dizziness and vertigo are among the most frequently encountered symptoms in clinical practice, particularly in outpatient neurology and otolaryngology settings [1]. These symptoms affect nearly 20–30% of the general population at some point in their lives and are often multifactorial in origin [2]. While peripheral vestibular disorders account for a significant proportion of cases, a substantial number remain diagnostically unexplained or misclassified due to overlapping features between vestibular and neurological conditions [3]. Among the non-peripheral causes, vestibular migraine has emerged as a key diagnostic consideration, especially in younger and middle-aged adults, yet its recognition in routine clinical workflows remains suboptimal [4].

Vestibular migraine is characterized by recurrent episodes of vertigo or dizziness associated with a history of migraine, in the absence of structural inner ear pathology [5]. Vestibular migraine is formally recognized as a distinct clinical entity in both the International Classification of Headache Disorders, 3rd Edition (ICHD-3), and the consensus diagnostic criteria established by the Bárány Society. These criteria jointly define vestibular migraine based on symptom duration, migraine history, and the presence of migrainous features during vestibular episodes [6]. Despite the availability of these standardized guidelines, clinical presentation frequently overlaps with other vestibular disorders, which may contribute to diagnostic delays or misclassification in routine practice. The previous literature has identified common features such as motion sensitivity, prolonged dizziness episodes, photophobia, and phonophobia, yet standardized screening and diagnostic tools have not been uniformly adopted across settings [5,7,8]. Moreover, vestibular migraine disproportionately affects females during their productive years, further compounding the clinical and socioeconomic burden [9]. Vestibular disorders, including vestibular migraine, are known to significantly impair quality of life and are frequently associated with psychiatric symptoms such as anxiety and depression, further exacerbating the overall disease burden [9].

Despite increasing recognition of vestibular migraine in the academic literature, there remains limited understanding of its precise prevalence in targeted subpopulations, such as females presenting with dizziness in primary or tertiary care [5,7,8]. While population-based studies have reported the general prevalence of vestibular migraine in community samples [10,11], such studies do not focus specifically on women within clinical settings or quantify the real-world functional burden using standardized disability metrics. This study addresses that gap by examining prevalence, symptom severity, and work-related impact in a defined female clinical population. Moreover, while the disabling impact of migraine on general quality of life is well-established, the specific functional impairments associated with vestibular migraine—including its effect on daily activities, work productivity, and self-perceived health status—have not been adequately quantified in real-world clinical cohorts [12]. Available studies have either focused on the diagnostic criteria or the pathophysiological overlap with migraine, with few integrating a comprehensive assessment of functional outcomes such as absenteeism, activity impairment, or dizziness-related disability [13].

This gap in the literature restricts the ability of clinicians to stratify patients based on symptom burden and hinders the development of evidence-based management algorithms. Given the individualized nature of vestibular migraine symptomatology and its psychosocial correlates, a personalized diagnostic and management approach is critical for optimizing clinical outcomes.

Given these considerations, the present study was designed with two specific objectives: (1) to assess the frequency and diagnostic rate of vestibular migraine among female patients aged 20–50 years presenting with dizziness and (2) to evaluate the impact of vestibular migraine on quality of life and work productivity using validated tools such as the Dizziness Handicap Inventory (DHI) and Work Productivity and Activity Impairment (WPAI) scale. It was hypothesized that vestibular migraine would be both highly prevalent and significantly associated with increased dizziness-related disability, poorer health perception, and greater functional impairment compared to non-migraine cases.

## 2. Materials and Methods

### 2.1. Design, Settings, and Ethics

This cross-sectional, observational study was conducted between June 2024 and April 2025 in the King Khalid University Medical City Hospital, Kingdom of Saudi Arabia. Ethical approval for this study was obtained from the institutional ethics committee at King Khalid University (approval no.: ECM#2025-712). All participants provided written informed consent prior to enrollment and this study was conducted in strict accordance with the ethical principles outlined in the Declaration of Helsinki.

### 2.2. Sample Size Estimation

A priori sample size calculation was conducted using G*Power (v3.1), based on a medium effect size (Cohen’s d = 0.5), power = 0.80, and α = 0.05, indicating a required minimum sample size of 128. Thus, the final sample of 196 exceeded the required threshold, ensuring adequate statistical power.

### 2.3. Participants

Female patients aged 20 to 50 years who presented with complaints of dizziness or vertigo were recruited during routine clinical consultations and underwent a comprehensive evaluation by a multidisciplinary team comprising otolaryngologists and neurologists. A total of 196 participants meeting the inclusion criteria were enrolled in this study following the provision of informed consent. The diagnosis of vestibular migraine was established based on the criteria outlined in the International Classification of Headache Disorders, 3rd edition (ICHD-3) [14], which includes at least five episodes of vestibular symptoms of moderate to severe intensity lasting 5 min to 72 h, a current or past history of migraine with or without aura, and at least 50% of vestibular episodes accompanied by migrainous features such as headache, photophobia, phonophobia, or visual aura (Table 1).

Inclusion criteria comprised females aged between 20 and 50 years reporting dizziness or vertigo as a primary symptom and willing to provide written informed consent. The decision to include only female participants was based on epidemiological evidence indicating a markedly higher prevalence and symptom burden of vestibular migraine in women, particularly during the reproductive age range. This focus allowed for a more precise evaluation of the condition’s functional and psychosocial impact within the most affected demographic subgroup. Participants were included irrespective of the duration of symptoms, provided that they were clinically stable and able to complete the required assessments. Exclusion criteria included individuals with confirmed peripheral vestibular disorders such as benign paroxysmal positional vertigo (BPPV), Ménière’s disease, vestibular neuritis, or any structural inner ear pathology diagnosed through clinical and audiovestibular examination. Additional exclusions included those with central nervous system lesions, pregnancy, chronic neurological conditions unrelated to migraine, and cognitive or psychiatric disorders that could interfere with informed consent or questionnaire completion. All selected participants underwent detailed clinical history-taking, physical examination, and appropriate diagnostic investigations to ensure accurate classification. Participants were recruited through consecutive non-probability sampling during outpatient visits, which may have introduced some selection bias. The non-migraine group comprised participants who reported dizziness or vertigo but did not meet the ICHD-3 diagnostic criteria for vestibular migraine following structured assessment. All individuals in the non-migraine group underwent complete audio-vestibular evaluation to exclude alternative vestibular disorders. Those with confirmed diagnoses of BPPV, Ménière’s disease, vestibular neuritis, persistent postural–perceptual dizziness (PPPD), or other identifiable central or peripheral conditions were excluded from this study. The remaining participants were classified as having idiopathic dizziness without a specific vestibular or neurological diagnosis.

### 2.4. Outcomes

The primary outcomes of this study were the diagnosis of vestibular migraine, dizziness-related disability, and work productivity impairment.

#### 2.4.1. Vestibular Migraine Diagnosis

Diagnosis was made by otolaryngologists and neurologists after structured clinical assessment and exclusion of peripheral vestibular disorders using audiological and vestibular investigations, including pure tone audiometry and videonystagmography.

##### Neurotological Examination

Neurotological assessment included a comprehensive bedside examination and instrumented vestibular testing conducted by a neurotologist. Standardized procedures followed Bárány Society guidelines, including head impulse testing, positional maneuvers, and videonystagmography (VNG) [15]. VNG protocols involved ocular motor evaluations (saccades, smooth pursuit, optokinetics), positional testing (Dix–Hallpike and supine roll tests), and bithermal caloric irrigation using air to assess lateral semicircular canal function [15]. Although air caloric testing is less precise than water irrigation and lacks established sex-based normative data, it was selected for practical feasibility. The Video Head Impulse Test (vHIT) was not included in the standard evaluation protocol. Similarly, orthoptic or formal neuro-ophthalmological testing was not performed as this study focused on neurotological assessment within an outpatient clinical setting. Findings were interpreted by experienced specialists to differentiate central from peripheral vestibular disorders.

##### Audiological Examination

Audiological testing was performed in a sound-treated environment using ANSI S3.6-calibrated two-channel audiometers [16]. Pure tone audiometry (air and bone conduction thresholds) covered frequencies from 250 Hz to 8000 Hz. Speech audiometry included speech reception thresholds and word recognition scores [16]. Tympanometry and acoustic reflex thresholds were measured to evaluate middle ear status. These assessments helped exclude patients with sensorineural hearing loss patterns suggestive of Ménière’s disease or other structural pathologies [16].

#### 2.4.2. Dizziness Handicap Inventory (DHI) Score

Dizziness-related disability was measured using the Dizziness Handicap Inventory (DHI), a 25-item validated tool that assesses the physical, emotional, and functional impact of dizziness [13]. Total DHI scores were interpreted as follows: 0–30, indicating mild handicap; 31–60, indicating moderate handicap; and 61–100, indicating severe handicap. This classification enabled symptom severity stratification among participants [13]. The DHI has been previously validated in Arabic-speaking populations, demonstrating good internal consistency and construct validity for assessing dizziness-related disability [17].

#### 2.4.3. Work Productivity and Activity Impairment (WPAI) Score

Work productivity impairment was evaluated using the Work Productivity and Activity Impairment Questionnaire–General Health (WPAI-GH v2.0) [18], which assessed absenteeism, presenteeism, overall work impairment, and activity limitation over the past seven days. WPAI scores were calculated according to the instrument guidelines and expressed as percentages, with higher percentages representing more significant impairment. All participants completed the activity impairment domain, and only employed participants completed the work-related sections.

#### 2.4.4. Demographic and Clinical Variables

Demographic information included age, recorded as a continuous variable in years, and body mass index (BMI), calculated using measured height and weight (kg/m^2^). Clinical history variables included motion sickness (yes/no), family history of migraine in first-degree relatives (yes/no), and history of anxiety or depression (yes/no), determined through structured interviews and verified with medical records when available. Comorbid medical conditions such as diabetes mellitus and hypertension were also recorded as binary variables and confirmed through clinical documentation. Both type 1 and type 2 diabetes mellitus were included without differentiation as this study focused on the presence of diagnosed diabetes mellitus as a comorbid condition.

#### 2.4.5. Vestibular and Migraine Features

The duration of dizziness or vertigo episodes was classified into three predefined categories: less than 5 min, 5 to 30 min, and more than 30 min, based on self-reported symptom patterns. In addition to episode duration, the presence of associated vestibular migraine symptoms—including pulsatile headache, photophobia, phonophobia, and visual aura—was systematically assessed during structured clinical interviews to meet ICHD-3 diagnostic criteria. The number of migraine episodes per month was recorded as a continuous variable, reflecting the patient’s recall of migraine frequency over the preceding four weeks. Where available, symptom diaries were used to support self-reported frequency.

#### 2.4.6. Health Status and Sleep Quality

Self-perceived health status was evaluated using a 4-point ordinal scale with options “poor”, “fair”, “good”, and “very good”. Sleep quality was assessed using the Pittsburgh Sleep Quality Index (PSQI), a widely used tool that evaluates sleep disturbance over a one-month interval [15]. The PSQI global score was calculated by summing seven component scores (sleep quality, latency, duration, efficiency, disturbances, use of medication, and daytime dysfunction), each scored from 0 to 3. A total global score >5 was interpreted as indicative of poor sleep quality. The PSQI global score ranges from 0 to 21, with higher scores indicating poorer sleep quality; a score above five was considered indicative of clinically significant sleep disturbance [19].

#### 2.4.7. Medication Use

Medication usage was recorded as a binary variable indicating whether the participant had used vestibular suppressants (betahistine, meclizine) or migraine-specific medications (e.g., flunarizine, propranolol, triptans) within the past month. All data were collected during a single clinical visit using standardized data collection instruments, with quality control checks performed by trained research staff to ensure completeness and accuracy. Participants were assessed prior to the initiation of any targeted treatment for vestibular migraine. No prophylactic or abortive migraine therapies were started or modified during the data collection period, allowing for the evaluation of baseline symptom burden and its functional impact without the confounding effects of active medical intervention.

### 2.5. Data Analysis

Continuous variables such as age, BMI, DHI score, WPAI score, and number of migraine episodes per month were summarized as means and standard deviations and group comparisons were conducted using independent sample t-tests or one-way ANOVA where appropriate. Categorical variables, including vestibular migraine diagnosis, family history of migraine, comorbidities, and medication use, were analyzed using chi-square tests. Pearson correlation coefficients were used to examine the relationship between quality-of-life measures (DHI, WPAI) and continuous predictors such as migraine frequency, sleep quality, and BMI. A multiple linear regression model was employed to identify independent predictors of work productivity impairment (WPAI score), with variables such as vestibular migraine diagnosis, DHI score, sleep quality, and anxiety/depression history entered as covariates. All tests were two-tailed, and a *p*-value of <0.05 was considered statistically significant. Data were checked for normality using Shapiro–Wilk tests and histograms, and parametric methods were deemed appropriate based on the results. Data analysis was performed using IBM SPSS Statistics version 24 (IBM Corporation, Armonk, NY, USA). Missing data were minimal (<5%) and handled using pairwise deletion. Missing data were assessed using Little’s MCAR test. Cases with >10% missing values in primary outcomes were excluded. For remaining variables with <5% missing, mean imputation was employed.

## 3. Results

Participants diagnosed with vestibular migraine demonstrated significantly higher rates of motion sickness, family history of migraine, and anxiety or depression compared to those without vestibular migraine (Table 2). Additionally, they were more likely to experience prolonged dizziness episodes exceeding 30 min and reported a higher frequency of migraine episodes per month. No significant differences were observed between the groups in terms of age, BMI, caffeine use, or comorbidities such as diabetes mellitus and hypertension.

Among female patients presenting with dizziness, the prevalence of vestibular migraine was 42.86%, with a 95% CI ranging from 36.13% to 49.86%, indicating a notably higher diagnostic yield than previously assumed (Table 3). This diagnostic rate was significantly greater than the expected population proportion of 20%, as confirmed by a one-sample z-test (z = 6.47, *p* < 0.001), suggesting that vestibular migraine may be underdiagnosed in routine clinical settings.

Participants with vestibular migraine experienced significantly longer dizziness episodes, with a mean duration of 37.62 ± 11.34 min (range: 10–65; IQR: 30–45), compared to 24.58 ± 10.49 min (range: 5–48; IQR: 15–30) in the non-migraine group. They also reported a markedly higher monthly migraine frequency (4.63 ± 1.97 vs. 1.78 ± 1.14 episodes; *p* < 0.001) (Table 4). The non-migraine group’s reported headache episodes did not fulfill the ICHD-3 criteria for migraine or vestibular migraine and likely reflect isolated, nonspecific headaches without migrainous features. This distinction was ensured through structured clinical evaluation by neurologists and otolaryngologists. Additionally, a greater proportion of vestibular migraine participants reported a history of motion sickness (66.67% vs. 31.25%, *p* = 0.001) and family history of migraine (59.52% vs. 24.11%, *p* = 0.002). There were no statistically significant differences in the prevalence of comorbid diabetes or hypertension between the groups.

**Figure 1:**
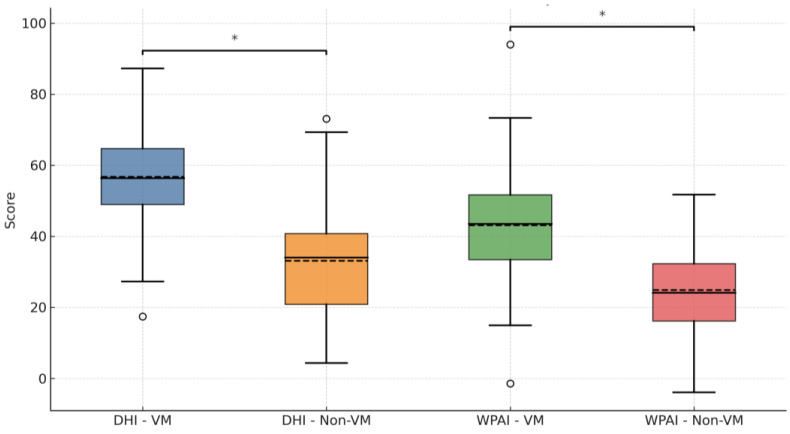
Boxplot comparison of Dizziness Handicap Inventory (DHI) and Work Productivity and Activity Impairment (WPAI) scores between vestibular migraine (VM) and non-migraine groups. Abbreviations: DHI = Dizziness Handicap Inventory; WPAI = Work Productivity and Activity Impairment; VM = vestibular migraine. “*” (asterisk)—Marks a statistically significant difference between the two compared groups (*p* < 0.05). “o” (circle)—Represents an outlier value, defined as a data point lying more than 1.5 times the interquartile range (IQR) above the third quartile or below the first quartile.

Participants diagnosed with vestibular migraine reported significantly higher levels of dizziness-related disability and work impairment, as evidenced by elevated mean DHI scores (58.34 ± 15.62 vs. 32.76 ± 14.83; *p* < 0.001) and markedly increased WPAI scores (42.19 ± 13.45 vs. 23.47 ± 12.90; *p* < 0.001) compared to non-migraine participants (Figure 1). These findings underscore the substantial burden that vestibular migraine imposes on daily functioning and occupational productivity.

**Figure 2:**
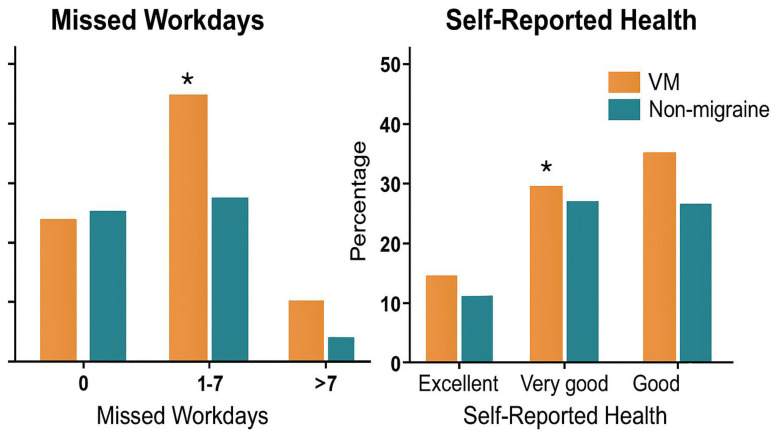
Comparison of missed workdays and self-reported health status between participants with vestibular migraine (VM) and non-migraine (NM) individuals. The left panel illustrates the number of workdays missed in the past month, while the right panel displays the distribution of subjective health ratings across groups. “*” (asterisk)—Denotes a statistically significant difference between VM and NM groups (*p* < 0.05).

Participants with vestibular migraine reported a significantly higher number of missed workdays in the past month (3.78 ± 2.14) compared to those without migraine (1.62 ± 1.45; *p* < 0.001), indicating greater occupational disruption (Figure 2). Moreover, poorer self-rated health status was more prevalent in the vestibular migraine group, with 27.38% rating their health as “poor” versus 8.93% in the non-migraine group (*p* = 0.002) and fewer individuals reporting “very good” health (8.33% vs. 23.21%; *p* = 0.018). These differences suggest a notable impact of vestibular migraine on perceived well-being and productivity.

**Figure 3:**
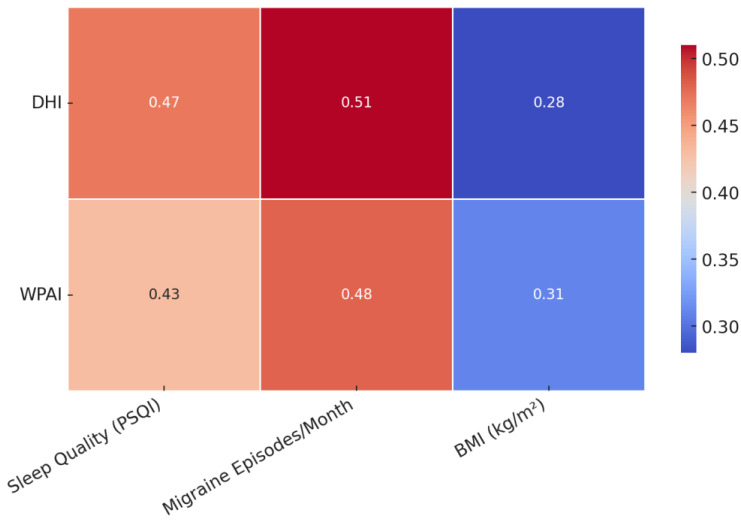
Heatmap of correlation between DHI/WPAI scores and sleep quality, migraine frequency, and BMI.

Significant positive correlations were observed between both DHI and WPAI scores and clinical variables. For DHI, the correlation with migraine frequency was *r* = 0.51, *p* < 0.001; that with sleep quality was *r* = 0.44, *p* < 0.001; and that with BMI was *r* = 0.31, *p* = 0.002. Similarly, WPAI scores were positively correlated with migraine frequency (*r* = 0.48, *p* < 0.001), sleep quality (*r* = 0.39, *p* < 0.001), and BMI (*r* = 0.27, *p* = 0.004), indicating that worse sleep, more frequent migraines, and higher BMI were associated with greater dizziness-related disability and work impairment (Figure 3). Migraine episodes per month showed the strongest correlation with DHI (*r* = 0.51, *p* < 0.001) and WPAI (*r* = 0.48, *p* < 0.001), followed by sleep quality and BMI, all reaching statistical significance.

Multiple linear regression analysis identified vestibular migraine diagnosis (B = 5.47, *p* < 0.001), anxiety or depression (B = 4.22, *p* < 0.001), poor sleep quality (B = 1.63, *p* = 0.006), higher BMI (B = 0.28, *p* = 0.013), and medication use (B = 1.35, *p* = 0.045) as significant independent predictors of increased work productivity impairment (Table 5). Age did not show a statistically significant association with WPAI scores, indicating that functional impairment in this context is more strongly driven by clinical and psychosocial factors than by age alone.

## 4. Discussion

This study aimed to evaluate the prevalence and diagnostic rate of vestibular migraine among females presenting with dizziness and examine its impact on quality of life and work productivity. The findings revealed that vestibular migraine was a relatively common diagnosis in this population, with a significantly higher rate than expected. Unlike prior epidemiological research based on general population surveys, the present study emphasizes a tertiary care cohort and highlights not only diagnostic prevalence but also the functional consequences of vestibular migraine in working-age women. By incorporating validated outcome measures such as the DHI and WPAI, this study advances the literature by linking diagnostic findings to real-world disability and productivity loss, thus supporting the need for more targeted diagnostic and interventional strategies in affected female populations. Clinical features such as prolonged dizziness episodes, frequent migraines, and a higher prevalence of motion sickness and family history of migraine were more prominent in those diagnosed with vestibular migraine. Furthermore, individuals with vestibular migraine exhibited greater dizziness-related disability, higher work impairment, and increased absenteeism, along with poorer self-perceived health status. Correlation analyses indicated that symptom burden and productivity loss were associated with poor sleep quality, migraine frequency, and higher BMI, while regression analysis identified vestibular migraine, psychological comorbidity, sleep disturbance, and medication use as significant predictors of work-related functional impairment.

The significantly higher diagnostic rate of vestibular migraine observed among females presenting with dizziness may be attributed to a combination of clinical characteristics that distinguish this condition from other vestibular disorders [16]. Hormonal fluctuations, particularly those related to the menstrual cycle, pregnancy, and perimenopause, are known to modulate migraine activity and may contribute to the disproportionately higher prevalence of vestibular migraine in women [20]. Estrogen withdrawal has been implicated in increasing neuronal excitability and central sensitization, which could amplify vestibular symptomatology in susceptible individuals [20]. In addition to hormonal influences, the marked female preponderance in VM may also stem from sex-related differences in vestibular sensitivity, as demonstrated in responses to caloric stimulation and subjective visual vertical testing [21]. Higher physiological reactivity in the female vestibular system could predispose women to more symptomatic manifestations [20]. Sociocultural dynamics—such as greater health-seeking behavior among women—may further contribute to higher diagnostic rates, while underdiagnosis in men cannot be ruled out. This pattern is supported by epidemiological studies indicating that women are more likely than men to seek medical care for dizziness, migraine, and other somatic symptoms, potentially influencing diagnostic prevalence rates [21]. Limited male representation in the present study reflects the higher observed prevalence in females but also highlights the need for future investigations to explore potential sex-based diagnostic disparities and sensory processing differences.

Prolonged dizziness episodes, frequent migraine attacks, and a prominent history of motion sickness suggest a distinct neurophysiological profile marked by central sensitization and altered vestibular processing [22]. Multiple studies have consistently shown a higher prevalence of motion sickness susceptibility among individuals with vestibular migraine, suggesting overlapping patho-mechanisms involving heightened vestibular responsiveness and impaired sensory integration [23]. This association is thought to arise from dysfunction in multisensory convergence pathways—particularly within the brainstem and cerebellum—that mediate both vestibular and visuo-motion inputs [24]. As such, motion sensitivity may serve as a clinically useful marker in distinguishing VM from other episodic vertigo syndromes and supports the inclusion of motion sickness history in diagnostic evaluations [24]. The elevated prevalence of family history of migraine and co-existing anxiety or depression further underscores the potential genetic and psychosomatic underpinnings of vestibular migraine [25]. These features likely increase the clinical recognition of the condition when assessed systematically. Importantly, the absence of significant differences in BMI, age, or comorbid cardiovascular conditions implies that vestibular migraine is more closely associated with neurological and functional symptom patterns than metabolic or age-related factors [26]. These findings are in line with previous research highlighting the diagnostic complexity and frequency of vestibular migraine in otoneurological clinics [5,27,28]. Given the overlapping symptomatology, particularly episodic vertigo and auditory disturbances, Ménière’s disease remains a critical differential diagnosis of vestibular migraine. High-resolution magnetic resonance imaging (MRI) of the inner ear, particularly delayed intravenous gadolinium-enhanced FLAIR sequences, can assist in identifying endolymphatic hydrops and ruling out structural anomalies, thereby supporting the differentiation of vestibular migraine from Ménière’s disease and other central vestibular disorders. Distinguishing between the two conditions often requires a comprehensive audiovestibular assessment, as both can present with fluctuating vertigo and associated sensory symptoms, leading to diagnostic ambiguity in clinical practice [29]. Shen et al. [5] reported a similar overrepresentation of migraine history and motion sensitivity in patients with vestibular complaints, supporting the relevance of these features in clinical differentiation [5]. Similarly, Haro Hernández et al. [20] emphasized the importance of episodic vertigo and migrainous features in refining diagnostic accuracy. The association between vestibular migraine and psychiatric comorbidities has also been corroborated by Mallampalli et al. [27], who noted elevated anxiety and depressive symptoms in affected patients. The convergence of these data reinforces the validity of the current findings and underscores the need for heightened clinical awareness to facilitate timely diagnosis and intervention.

The markedly elevated DHI and WPAI scores observed among participants with vestibular migraine reflect the substantial impact of this condition on both perceived disability and occupational functioning [30]. The dizziness-related handicap captured by the DHI likely results from recurrent episodes of vertigo, imbalance, and associated sensory disturbances, which can severely disrupt daily tasks and social participation [31]. Similarly, the impairment in work productivity measured by the WPAI can be attributed to symptom unpredictability, heightened sensory sensitivity, and cognitive disturbances commonly reported in migraine disorders [32]. These limitations may be further compounded by comorbid psychological factors such as anxiety and sleep disturbances, which are frequently observed in this population and may intensify subjective burden. The current findings align with the existing literature documenting functional limitations in individuals with vestibular migraine. Smith et al. [33] described how vestibular dysfunction coupled with migraine pathophysiology leads to significant impairment in spatial orientation and postural control, which are critical for daily functioning [33]. Batinović et al. [34] reported that vestibular migraine patients score significantly higher on dizziness-related disability scales compared to those with other vestibular disorders. Furthermore, Ford et al. [32] demonstrated that such patients frequently experience work absenteeism and reduced productivity, echoing the elevated WPAI scores found in this study. These corroborative findings reinforce the understanding of vestibular migraine as a condition with profound implications on both quality of life and functional capacity.

### 4.1. Clinical Significance of This Study

The clinical significance of this study lies in its identification of vestibular migraine as a prevalent and underrecognized cause of dizziness in females aged 20–50, with substantial implications for both diagnostic accuracy and patient outcomes. By delineating specific clinical features—such as prolonged dizziness, frequent migraine episodes, motion sickness history, and psychiatric comorbidities—this study provides actionable markers that can improve early identification and differentiation of vestibular migraine from other vestibular disorders. Moreover, the demonstrated impact on quality of life and work productivity, as quantified by DHI and WPAI scores, highlights the necessity for timely diagnosis and multidisciplinary management. These findings support the integration of vestibular migraine screening into routine dizziness assessments in clinical practice, ultimately facilitating more targeted interventions and reducing the burden of functional impairment in this population.

### 4.2. Limitations of This Study

This study is limited by its cross-sectional design, which precludes causal inferences regarding the relationship between vestibular migraine and associated functional impairments. The reliance on self-reported measures such as the DHI, WPAI, and migraine frequency may introduce recall bias, while the exclusive inclusion of female participants aged 20–50 restricts the generalizability of findings to other demographic groups. Furthermore, as a single-center study from a tertiary-care university hospital, external validity may be limited due to referral bias and demographic homogeneity. Additionally, the absence of neuroimaging or electrophysiological validation limits the ability to correlate clinical findings with underlying pathophysiological mechanisms. Future research should incorporate longitudinal designs to assess the progression and treatment response of vestibular migraine, explore gender- and age-related differences, and integrate objective biomarkers to enhance diagnostic precision and therapeutic monitoring.

## 5. Conclusions

This study concludes that vestibular migraine is a common and clinically distinct diagnosis among females aged 20–50 presenting with dizziness, characterized by longer and more frequent dizziness episodes, a higher prevalence of motion sickness and family history of migraine, and greater psychosomatic comorbidity. The condition is associated with significantly higher levels of dizziness-related disability and work impairment, as reflected by elevated DHI and WPAI scores, increased absenteeism, and poorer self-rated health status. Moreover, clinical variables such as migraine frequency, sleep quality, BMI, and psychological history were significantly correlated with functional burden and identified as independent predictors of work productivity loss. These findings emphasize the need for targeted clinical recognition and management of vestibular migraine in this specific patient population.

## Figures and Tables

**Table 1:** Diagnostic criteria for vestibular migraine (ICHD-3).

Criterion	Description
A	At least five episodes fulfilling criteria B and C
B	Vestibular symptoms of moderate or severe intensity lasting between 5 min and 72 h
C	Current or past history of migraine with or without aura
D	At least 50% of vestibular episodes are associated with at least one of the following migrainous features:
Headache with at least two of the following: unilateral location, pulsating quality, moderate/severe pain intensity, aggravation by routine physical activity	
Photophobia and phonophobia	
Visual aura	
E	Not better accounted for by another vestibular or ICHD-3 diagnosis

**Table 2:** Demographic and clinical characteristics of participants.

Variable	Vestibular Migraine (*n* = 84)	Non-Migraine (*n* = 112)	*p*-Value
Age (years)	34.52 ± 7.21	35.17 ± 6.89	0.528
BMI (kg/m^2^)	25.48 ± 3.76	24.92 ± 4.10	0.317
Caffeine use (moderate/high)	52/32	68/44	0.491
History of motion sickness (%)	66.7	31.3	0.001
Family history of migraine (%)	59.5	24.1	0.002
Diabetes (%)	14.3	17.0	0.582
Hypertension (%)	20.2	23.2	0.391
Anxiety/depression history (%)	33.3	19.6	0.011
Duration of dizziness episodes < 5 min (%)	9.5	26.8	0.002
Duration of dizziness episodes 5–30 min (%)	39.3	42.9	0.734
Duration of dizziness episodes > 30 min (%)	51.2	30.4	0.006
Number of migraine episodes/month	4.63 ± 1.97	1.78 ± 1.14	<0.001

BMI: body mass index. Note: Statistical comparisons were made using independent sample *t*-tests for continuous variables and chi-square tests for categorical variables.

**Table 3:** Frequency and diagnostic rate of vestibular migraine among females with dizziness.

Parameter	Value
Vestibular migraine diagnosis (Yes)	84 (42.86%)
Vestibular migraine diagnosis (No)	112 (57.14%)
Prevalence rate (95% CI)	42.86% (36.13–49.86%)
Diagnostic proportion	42.86%
Z-test for expected proportion (20%)	z = 6.47, *p* = 0.000

CI: confidence interval; Z: z-statistic; %: percentage.

**Table 4:** Comparison of clinical features in participants with and without vestibular migraine.

Variable	Vestibular Migraine (*n* = 84)	Non-Migraine (*n* = 112)	*p*-Value
Duration of dizziness episodes (min)	37.62 ± 11.34	24.58 ± 10.49	0.032
Number of migraine episodes/month	4.63 ± 1.97	1.78 ± 1.14	<0.001
History of motion sickness (%)	66.67	31.25	0.001
Family history of migraine (%)	59.52	24.11	0.002
Comorbid diabetes (%)	14.29	17.05	0.582
Comorbid hypertension (%)	20.24	23.21	0.391

**Table 5:** Multiple linear regression: predictors of work impairment (WPAI Score).

Predictor Variable	B (Unstandardized Coefficient)	Standard Error	95% CI (Lower–Upper)	*p*-Value
Age (years)	0.12	0.09	−0.05–0.29	0.141
BMI (kg/m^2^)	0.28	0.11	0.06–0.50	0.013
Vestibular Migraine Diagnosis (Yes = 1)	5.47	1.42	2.67–8.27	<0.001
Poor Sleep Quality (PSQI score)	1.63	0.59	0.48–2.78	0.006
Anxiety/Depression (Yes = 1)	4.22	1.12	2.01–6.43	<0.001
Medication Use (Yes = 1)	1.35	0.67	0.03–2.67	0.045

WPAI: Work Productivity and Activity Impairment; B: unstandardized coefficient; CI: confidence interval; PSQI: Pittsburgh Sleep Quality Index; BMI: body mass index.

## Data Availability

The data presented in this study are openly available in Zenodo at https://doi.org/10.5281/zenodo.15845705. You can cite all versions by using the https://doi.org/10.5281/zenodo.15845704.

## Abbreviations

DHI	Dizziness Handicap Inventory
ICHD-3	International Classification of Headache Disorders, 3rd Edition
PSQI	Pittsburgh Sleep Quality Index
VNG	Videonystagmography
VM	Vestibular Migraine
WPAI	Work Productivity and Activity Impairment
